# Should length of stay in hospital be the endpoint in arthroplasty?

**DOI:** 10.1080/17453674.2020.1763570

**Published:** 2020-05-12

**Authors:** Lars Nordsletten

**Affiliations:** Division of Orthopaedic Surgery, Oslo University Hospital, Oslo, Norway

This is, interestingly, one of the 10 most cited papers in the history of Acta after year 2000 (Husted et al. 2011). Interestingly, since length of stay (LOS) is not the most important parameter in arthroplasty: freedom of pain, normalized function and longevity are the ultimate goals. Why is then LOS of such interest? Hospital beds are a limited resource in many parts of the world, irrespective of payer system. LOS has therefore come under surveillance, to the degree that day care arthroplasty has become common in certain hospitals (Hartog et al. 2015). Remember that it is not more than 15 years ago since patients stayed in hospital for 1 to 2 weeks after total joint arthroplasty (TJA).

The study on 207 patients undergoing hip or knee arthroplasty registered 2 times a day whether fulfillment of each of the discharge criteria had been obtained, and detailed reason(s) for not being discharged. Husted et al. found that in a fast track system pain, dizziness, and general weakness were the main reasons for not being discharged after 24 and 48 hours in 80% of patients. Median LOS was 2 days, and 95% were discharged after 3 days. Waiting for blood transfusion, start of physiotherapy, and for postoperative radiographic examination delayed the discharge for 20%. The first factors can be seen as patient related, while the last ones are hospital factors. The hospital factors could be organizationally removed, while patient factors probably could not be changed. The authors had previously shown that readmissions were not increased by the fast-track system. The authors themselves concluded that the findings offered the possibility of safe reduction of LOS after fast-track hip or knee arthroplasty.

Now, nearly 10 years after its publication, it can be discussed whether being highly cited is equivalent to being an important scientific paper? The study was non-selective in including all patients scheduled for TJA in a 6 months period, thereby it was valid to all patients treated at Hvidovre hospital, and maybe to all patient in Denmark and Scandinavia. It was published in a period when LOS was rapidly decreasing due to implementation of fast-track surgery around the world. Husted et al. studied why some patients were in hospital while others had returned home, a topic which interested all researchers in hospital logistics and post-operative analgesia. The 176 citing papers are mostly on rapid recovery and analgesia. The study reached a peak with 18 citations in 2018. The most surprising citation was in pediatric urology, but also that study was on enhanced recovery after surgery (Haid et al. 2020).

Husted and Kehlet have been the pioneers in rapid recovery in Scandinavia, with numerous publications on analgesia (which is a prerequisite for rapid discharge), and recently outpatient total joint surgery (Gromov et al. 2019). The value of the 2011 paper has perhaps been mostly to pave the way for this unthought possibility just 15 years ago, leaving hospital with a new hip or knee the same day as you went in through the hospital doors.
